# Focus on P2X7R in microglia: its mechanism of action and therapeutic prospects in various neuropathic pain models

**DOI:** 10.3389/fphar.2025.1555732

**Published:** 2025-03-25

**Authors:** Kai Zhang, Rui Ran, Cheng-Jun Zhang, Linna Wang, Hai-Hong Zhang

**Affiliations:** ^1^ Department of Orthopedics, The Second Hospital of Lanzhou University, Lanzhou, China; ^2^ Orthopedics Key Laboratory of Gansu Province, Lanzhou, China; ^3^ Lanzhou Biotechnique Development Co., Ltd., Lanzhou, China

**Keywords:** neuropathic pain, P2X7R, microglia, M1/M2 polarization, cytokine, neuroinflammation

## Abstract

Neuropathic pain (NP) is a common symptom of many diseases and is caused by direct or indirect damage to the nervous system. Tricyclic antidepressants and serotonin-norepinephrine reuptake inhibitors are typical drugs used in clinical practice to suppress pain. However, these drugs have drawbacks, including a short duration of action, a limited analgesic effect, and possible dependence and side effects. Therefore, developing more effective NP treatment strategies has become a priority in medical research and has attracted much research attention. P2X7 receptor (P2X7R) is a non-selective cation channel activated by adenosine triphosphate and is mainly expressed in microglia in the central nervous system. Microglial P2X7R plays an important role in pain regulation, suggesting that it could be a potential target for drug development. This review comprehensively and objectively discussed the latest research progress of P2X7R, including its structural characteristics, functional properties, relationship with microglial activation and polarization, mechanism of action, and potential therapeutic strategies in multiple NP models. This study aimed to provide in-depth insights into the association between P2X7R and NP and explore the mechanism of action of P2X7R in the pathological process of NP and the translational potential and clinical application prospects of P2X7R antagonists in pain treatment, providing a scientific basis for the precise treatment of NP.

## 1 Introduction

Pain is an unpleasant sensory and emotional experience caused by actual or potential tissue damage (De Ridder et al., 2021). It is a warning indication of tissue damage and is transmitted from the periphery to the brain via a network of specialized receptors and nerve fibers (Mulè et al., 2024). Pain induced by pathological conditions such as cancer, infection, diabetes, and trauma is sometimes called neuropathic pain (NP) (Borges et al., 2021). In 2019, the International Association for the Study of Pain (IASP) updated the definition of NP to “pain caused by lesions or diseases of the somatic sensory nervous system” (Scholz et al., 2019). Neuropathic pain was previously defined as “pain triggered or caused by primary lesions, dysfunctions, or temporary disturbances of the peripheral or central nervous system” (Vervest and Schimmel, 1988). This definition incorporates two important changes: functional impairment and neuronopathy. Functional impairment is no longer recognized as a criterion under the revised definition. Consequently, it is believed that NP occurrence is associated with abnormalities in the pain transmission and perception of the nervous system. When neurons are damaged, their excitability increases, making them more susceptible to depolarization from even slight stimulation, leading to abnormal pain signal transmission (Koga et al., 2023). Neuropathic pain is commonly described as severe burning, stabbing, squeezing, or freezing pain (Pickering et al., 2017). These pain attacks can occur alone or alongside persistent pain, which is often unbearable for patients and seriously affects their quality of life (Torres et al., 2022). Currently, IASP recommends tricyclic antidepressants, serotonin-norepinephrine reuptake inhibitors, gabapentin, and pregabalin as first-line drugs for NP treatment (Finnerup et al., 2015). However, these drugs exhibit varying degrees of side effects, and the therapeutic effects are not ideal (Finnerup et al., 2015). As a result, developing new strategies or drugs that have minimal side effects and can achieve long-term effects with a single dose is an urgent need.

Following nerve injury, several molecular and cellular changes occur in the peripheral and central nervous systems. Among these, ATP and its purinergic receptors have been extensively studied. The concept of purinergic signaling was first proposed in 1972 (Burnstock, 1972), and purinergic receptors were defined in 1976 (Burnstock, 1976). Purinergic receptors are widely involved in immune responses, inflammation, and pain pathways. In 1993, the first P2 purinergic receptor was successfully cloned (Lustig et al., 1993). It is classified into two subtypes: ion-gated receptors (P2X receptors [P2XRs]) and G protein-coupled receptors (P2Y receptors [P2YRs]) (Kohno and Tsuda, 2021). The most characteristic mechanism of P2XR activity is their high permeability to Ca^2+^ (Khakh and North, 2012). Activation of P2XRs causes an increase in intracellular Ca^2+^ and a depolarization wave, resulting in signal transmission. Additionally, P2XR signaling involves functional interactions with other ion channels, including K^+^ efflux and Na^+^ influx (Khakh and North, 2012). The P2X7 receptor is of particular interest not only because it is expressed primarily in cells of the hematopoietic lineage and glial cells but also because it has a relatively low affinity for ATP, with maximal activity being achieved at ATP concentrations found under pathological conditions (100–1,000 µM). Its prolonged activation results in the formation of transient pores in the cell membrane, which are permeable to soluble substances up to 900 Da, potentially causing cytotoxicity (Abbracchio et al., 2009). Furthermore, it activates several intracellular signal transduction pathways, including mitogen-activated protein kinase (MAPK), calcineurin (CN), and nuclear factor-κβ (NF-κβ) (Skaper et al., 2010). Accordingly, this receptor may play a special role under pathological conditions. Moreover, P2X7Rs perform several physiological functions in the central nervous system, including cell proliferation, cell growth, neurotransmitter release, and neuron-glia communication (Skaper et al., 2010) (Sperlágh and Illes, 2014) (Tewari and Seth, 2015). Consequently, P2X7Rs are believed to play a key role in the occurrence and development of NP (Godoy et al., 2019).

Microglia are the primary glial cell type responsible for the occurrence and development of NP (Zhou et al., 2021) (Kim et al., 2021) (Yuan et al., 2023). They are the most common type of immune cells in the central nervous system (Borst et al., 2021). Microglia can regulate neuron excitability and inhibition, thereby regulating neuronal network activity (Villasana-Salazar and Vezzani, 2023). The mechanism of interaction between glial cells and neurons is complex. Activated microglia can polarize into M1 type (promoting inflammatory response and exerting cytotoxic effects) or M2 type (inhibiting inflammatory response and producing tissue protection effects) and release a variety of glial transmitters and cytokines, thereby affecting the excitability and sensory transmission of surrounding neurons (Zhang et al., 2024). This interaction may be crucial in the occurrence and maintenance of NP. Activation of P2X7R on microglia can increase calcium ions in microglia, increase microglial excitability, lead to phosphorylation of calmodulin-dependent protein kinase, and activate the synthesis and release of multiple cytokines, causing changes in the local inflammatory microenvironment (Martel-Gallegos et al., 2013). Microglial activation triggers the release of inflammatory factors such as interleukin-1β (IL-1β), IL-6, and tumor necrosis factor-α (TNF-α) (Yang et al., 2024). These mediators play a key role in the occurrence and maintenance of NP (Clark et al., 2006) (Zhang et al., 2018). Additionally, inhibiting P2X7R activation can inhibit the abnormal excitability and inflammatory response of microglia, thereby effectively alleviating the symptoms of NP (Tsuda, 2017) (Xie et al., 2017). Recently, more studies have revealed that microglia play an important role in neurodevelopment and neurodegenerative diseases. For example, microglia play a key role in the inflammatory response and neuroprotection in diseases such as Alzheimer’s disease, Parkinson’s disease, and spinal cord injury (Zhang et al., 2024) (Finneran et al., 2019) (Cignarella et al., 2020). However, the specific mechanism by which P2X7R acts on glial cells to cause and maintain NP is yet to be elucidated. Exploring the specific mechanism of action of P2X7R on microglia in NP is expected to lead to breakthroughs and treatment options for NP.

## 2 Pathological mechanism of neuropathic pain

Neuropathic pain is a condition caused by direct or indirect damage, dysfunction, or transient disorder of the central or peripheral nervous system. The pain sensation persists even after the noxious stimulus has been withdrawn. Nerve severance or damage, metabolic imbalance, viral infection, or an autoimmune response are all potential causes of NP. Symptoms include paroxysmal or persistent spontaneous pain, allodynia, and hypersensitivity to touch, cold, and heat (Ciapała and Mika, 2023). Many cases progress to chronic pain, and in many patients, they cause comorbidities of mood disorders such as anxiety and depression (Mokhtari and Uludag, 2024), causing significant suffering. According to recent statistics, the global incidence of chronic NP has reached 6.9%–10%, which has caused a significant burden on patients’ lives and socioeconomic status (Scholz et al., 2019). However, no effective clinical treatment strategy is currently available, and the commonly used treatment drugs are still opioids, antidepressants, and antiepileptic drugs. (Finnerup et al., 2015). These drugs are often ineffective for most patients—effective for a small number of patients and accompanied by side effects (Finnerup et al., 2021). Recently, with in-depth research on the mechanism of NP, its pathological process has been widely recognized. It mainly includes the following aspects: (De Ridder et al., 2021): Nociceptor sensitization and pain fiber induction: Nerve damage causes nerve fiber loss and axonal demyelination, resulting in abnormal pain signal transmission (Iwata et al., 2024). (Mulè et al., 2024) Activation of spinal microglia and release of inflammatory mediators: The activity of inflammatory factors and glial cells facilitates neuroinflammation, inhibits nerve regeneration, and alters pain thresholds (Xu et al., 2020). (Borges et al., 2021) Abnormal discharge of sensory afferent neurons: Neurons discharge abnormally following spinal cord injury, resulting in changes in low- and high-threshold mechanoreceptors (Boada et al., 2019). (Scholz et al., 2019) Neurotransmitter release: The effects of glutamatergic neurotransmitters and associated ion channels affect the transmission of pain signals (Alexander et al., 2022). (Vervest and Schimmel, 1988) Enhanced synaptic transmission: Enhanced synaptic function may aggravate pain (Ko et al., 2023). (Koga et al., 2023) Infiltration of inflammatory cells and release of inflammatory mediators in the injured area aggravate nerve damage and pain (Makabe et al., 2024). Overall, significant progress has been made in understanding the pathological mechanism of NP; however, its specific mechanism requires further in-depth research.

## 3 Overview of P2X7 receptor

As research into NP progresses, studies have discovered that P2X7R, which is widely distributed throughout the nervous system, plays a vital role in the occurrence and development of NP. Notably, pharmacological blockade of P2X7R-mediated microglial polarization has emerged as a promising therapeutic strategy for modulating NP.

### 3.1 Basic structure and characteristics of P2X7 receptor

**FIGURE 1:**
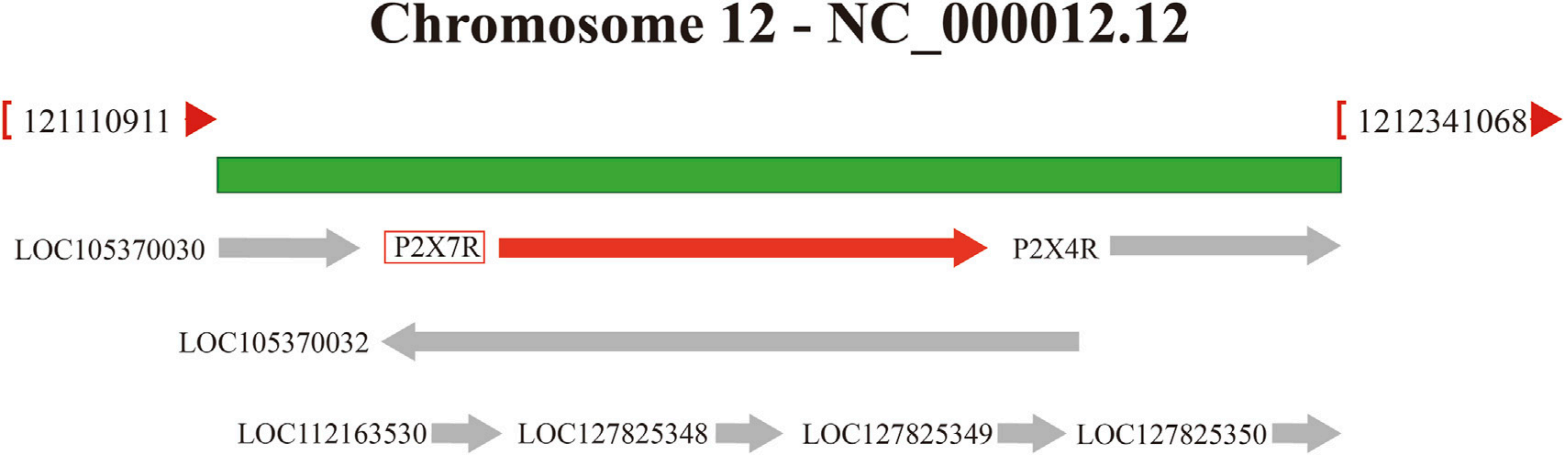
P2X7R gene is located on chromosome 12q24, next to the P2X4R gene.

The P2X7R gene is located on 12q24 and has a total length of approximately 50 kb, including 13 exons (Figure 1). Its mRNA is 3,155 bp long and comprises 595 amino acids. It has an amino-terminal (N-terminal), a carboxyl-terminal (C-terminal), an intracellular domain, and two transmembrane domains (TM1 and TM2) with conserved extracellular loops (Surprenant et al., 1996). The C-terminus of P2X7R is approximately 200 amino acids longer than other known P2XRs and is not present in other P2XRs (Surprenant et al., 1996). It also determines its unique physiological function. The P2X7R protein usually forms trimers on the cell membrane and sometimes exists as oligomers (McCarthy et al., 2019). The main physiological function of the P2X7R is to form ion channels and plasma membrane pores on the cell membrane surface, which aid in signal transduction, cytokine secretion, and cell proliferation (Sluyter, 2017). The activation of the P2X7R has a bidirectional effect under different stimulation conditions. When low concentrations of extracellular agonists (ATP) stimulate the P2X7R, it can quickly open a transmembrane ion channel that allows small cations such as Na^+^, K^+^, and Ca^2+^ to pass through, promoting the influx of Na^+^ and Ca^2+^ and the efflux of K^+^. Under long-term stimulation of high concentrations of agonists, the P2X7R will form non-selective plasma membrane pores, allowing molecules with a relative molecular mass of less than 900 Da to pass through, increasing the permeability of the cell membrane and ultimately inducing cell death (Di Virgilio et al., 2018). Importantly, ATP concentration influences presynaptic and postsynaptic P2X7R activation, thereby regulating the dynamics of pain signaling (Wu et al., 2022). In a healthy state, ATP levels are typically low, allowing P2X7R to remain physiologically inactive. Therefore, P2X7R is primarily activated when the endogenous ligand ATP concentration exceeds 100 μM (Huang et al., 2014). P2X7R targets the presynaptic terminals of the central and peripheral nervous systems. Activation of P2X7R promotes the release of norepinephrine, acetylcholine and other neurotransmitters from the presynaptic terminals (Deuchars et al., 2001), thereby increasing the excitability of the nervous system. Additionally, P2X7R regulates intercellular communication between neurons and glial cells, especially during inflammation. For example, microglia release exosomes after P2X7R activation, and the inflammatory signals contained within them are transmitted to neurons (Delpech et al., 2019). Furthermore, the activation of microglial P2X7R can cause P2X4 receptors to translocate to the surface and release factors such as IL-1β and derived nerve growth factor (BDNF), affecting neuron excitability and triggering pain perception (Trang et al., 2009).

### 3.2 Diverse localization of P2X7 receptor in the central nervous system

P2X7 receptor is a major driver of inflammation; it is widely expressed in cells of the innate and adaptive immune systems in animals and humans (Di Virgilio et al., 2018). P2X7 receptor is primarily localized in the microglia of the central nervous system, the resident macrophages in the brain (Bhattacharya and Jones, 2018). However, glial cells such as astrocytes and oligodendrocytes also express these receptors, albeit at lower densities (Illes et al., 2012) (Zhao et al., 2021). Importantly, it is closely associated with pain responses. P2X7 receptor participates in the extracellular signal-regulated kinase 1 (ERK1) signaling pathway in satellite glial cells to mediate pain responses and is regulated by transient receptor potential vanilloid receptors 1 (TRPV1) and 4 (TRPV4) (Fan et al., 2021) (Wang et al., 2021). Recently, Wu Y. et al. (2021) established a chronic constriction injury model and discovered a novel transcriptional mechanism in which BDNF promoted P2X7R expression in dorsal root ganglion neurons. When neuroinflammation occurs, P2X7R expression increases. P2X7 receptor inhibition can alleviate hyperalgesia and reduce astrocyte and microglial activation in the L5 dorsal horn (Hu et al., 2020). In summary, P2X7R is an important molecule that is widely expressed throughout the body and is particularly concentrated in microglia.

### 3.3 P2X7 receptor is involved in microglial activation

Microglia are the main immune cells resident in the central nervous system. They are macrophages abundant in the central nervous system and are significantly involved in the cellular immune process of the central nervous system (Fang and Lai, 2024). Microglia, namely the M0 type, are normally quiescent. In this state, they constantly sense environmental changes and respond to invading pathogens, toxins, and cell debris, acting as immune monitors (Chen et al., 2025). Activated microglia respond differently to external stimuli and act as a “double-edged sword.” Acutely activated microglia promote tissue repair by removing invading pathogens and cell debris, whereas persistently activated microglia induce chronic neuroinflammation and promote disease progression. Microglia in the central nervous system play a significant role in pain regulation owing to their unique functions (Du et al., 2020). P2X7 receptor-mediated microglial activation and polarization are often closely related to the occurrence, development, and regulation of inflammation, and these activation and polarization states can affect the inflammatory process (Dworsky-Fried et al., 2020). P2X7 receptor plays a key role in microglial activation (Fernandes et al., 2016). Its overexpression can increase microglial membrane permeability, boost microglial proliferation, promote inflammatory factor release, and cause further neuronal damage (Zhang et al., 2020). Activation of P2X7R can cause the cell membrane to form non-selective cation channels, allowing Na^+^ and Ca^2+^ influx and K^+^ efflux (Sperlágh and Illes, 2014; Di Virgilio et al., 2017). This increase in calcium influx alters the intracellular calcium ion balance, thereby affecting cell signal transduction and cytokine secretion. Additionally, P2X7R activation is associated with calcium-dependent signaling pathways, including the activation of phospholipase A2, phospholipase D, MAPK, and NF-κB (Chen et al., 2022; Carroll et al., 2009; Shiratori et al., 2010; Liu et al., 2011). The activation of these signaling pathways regulates microglial function and responsiveness.

### 3.4 P2X7 receptor is involved in microglial polarization

Microglia can polarize into two phenotypes in response to injury and inflammatory stimuli: M1, which is pro-inflammatory and damaging, and M2, which is anti-inflammatory and protective (Lan et al., 2017) (Scott et al., 2021) (Caffarel and Braza, 2022). Polarized M1-type microglia release pro-inflammatory factors and harmful mediators, aggravating inflammatory cell infiltration, whereas M2-type microglia reduce the inflammatory response by releasing anti-inflammatory factors (Atta et al., 2023). Acute and controlled activation of microglia promotes neuroprotection and neural repair, whereas chronic activation of microglia usually leads to excessive production of neurotoxic cytokines (M1 type polarization) and other inflammatory mediators (Au and Ma, 2022). These polarized microglia release different signaling molecules that exert different effects on target neurons to regulate pain. Inducers such as TNF-α, interferon-γ, and lipopolysaccharide cause microglia to polarize to the M1 phenotype (Xia et al., 2022), expressing specific markers such as CD86, CD32, and CD16 surface determinants, increasing the expression of inducible nitric oxide synthase (iNOS) and secreting pro-inflammatory cytokines such as IL-1β, IL-6, IL-18, and TNF-α (Higashi et al., 2017), exacerbating pain. M2 polarization is induced by IL-4 and transforming growth factor-β (Radpour et al., 2023) and is characterized by the expression of CD163 and CD206, upregulation of arginase-1, and secretion of IL-10, CNTF-1, IGF-1, and NGF-1, which help relieve pain (Si et al., 2024).

**FIGURE 2:**
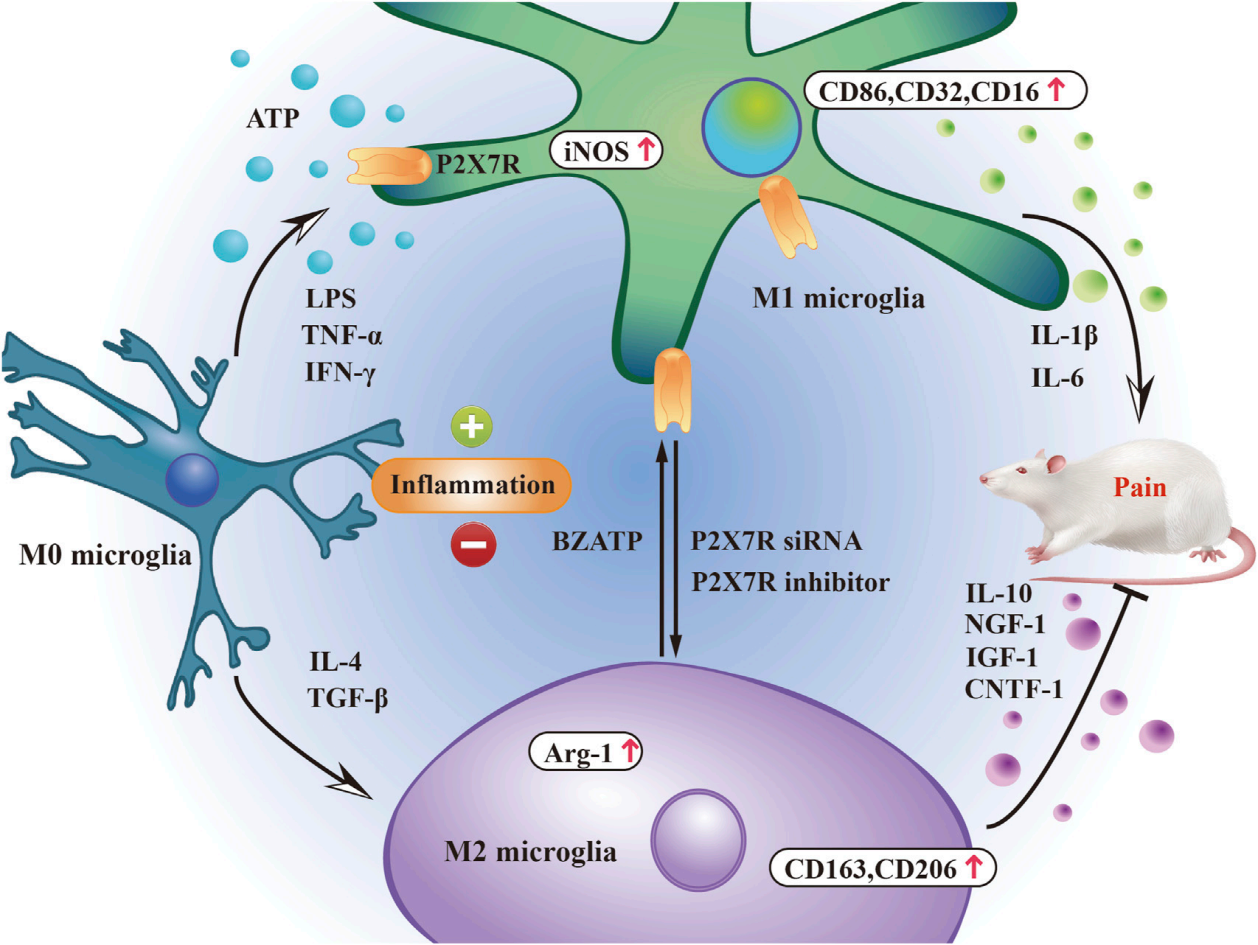
P2X7R participates in pain regulation by regulating microglial phenotype. The balance between pro- and anti-inflammatory mediators released by M1/M2 microglia is closely related to the pain state induced by P2X7R.

P2X7R activation is closely related to M1 microglia, and P2X7R is involved in the development and progression of microglia-mediated inflammation. P2X7 receptor-mediated Ca^2+^ influx is a key step in activating the NLRP3 inflammasome. The assembly of the NLRP3 inflammasome leads to the activation of caspase-1, which promotes the maturation and release of pro-inflammatory cytokines IL-1β and IL-18. These cytokines are important markers of M1 microglia and can further enhance neuroinflammatory responses (Ren and Illes, 2022). Additionally, P2X7R activation drives M1 polarization by promoting the activation of the NF-κB signaling pathway (Zhang et al., 2022). NF-κB activation results in the transcription of multiple pro-inflammatory genes, including TNF-α, IL-6, and iNOS, which are highly expressed in M1 microglia (Wu et al., 2022). Furthermore, P2X7R can be used as a potential molecular target for M1 microglial positron emission tomography imaging (Van Weehaeghe et al., 2019). P2X7 receptor regulates M1/M2 polarization of rat microglia in inflammation caused by brain injury and chronic sciatic nerve constriction injury (Gui et al., 2020) (Song et al., 2023). Small interfering RNA (siRNA)-mediated knockdown of P2X7R (Wu et al., 2022) or the use of P2X7R inhibitors (Gui et al., 2020) can effectively switch microglial polarization from M1 to M2, effectively alleviating pain. These indicate that P2X7R expression can influence the severity and appearance of pain by regulating microglial polarization direction (Figure 2).

## 4 Role of P2X7 receptor in neuropathic pain

P2X7 receptor may play a key role in pain and inflammation. Following sciatic nerve injury, P2X7R expression increases significantly, which is related to the occurrence of hyperalgesia (Yu et al., 2018). Sciatic nerve injury causes sensory nerve fibers to transmit signals to primary spinal cord centers, upregulating P2X7R expression in spinal microglia and inducing hyperalgesia (Masoodifar et al., 2021). Researchers have observed NP relief in many experimental models by inhibiting P2X7R using drugs such as A-438079, ox-ATP, or A-839977 (Zhou et al., 2023) (Bianco et al., 2012) (Honore et al., 2009). Upregulation of P2X7R expression and its activation in damaged nerves promotes IL-1β maturation and release, an upstream mechanism for pain signaling (Luchting et al., 2016). Clark et al. (2010) discovered a relationship between P2X7R activation and p-38/MAPK-dependent protease release in microglia and proposed that inhibiting these proteases may be another mechanism by which P2X7R antagonists alleviate NP. Additionally, P2X7R activation leads to the release of several diffusible factors from microglia, including TNF-α, iNOS, prostaglandin E2, cyclooxygenase 2, and BDNF, all of which are closely related to NP development (Inoue, 2017). P2X7 receptor-induced pore formation may also trigger a cascade of downstream effects that may be involved in the generation of pain hypersensitivity. Consequently, attenuating pore formation without interfering with cation channel activity may provide a new strategy for alleviating chronic pain (Sorge et al., 2012). Furthermore, P2X7R contributes to the development of tactile allodynia and nociceptive hypersensitivity in models of chronic constriction injury (CCI), spinal nerve ligation (SNL), diabetic neuropathic pain (DNP), and selective spinal nerve injury (SNI). The functional differences of P2X7 receptors in different neuropathic pain models are mostly reflected in the intensity and mechanism of their effects on microglial activation, neuronal excitability regulation, and inflammatory factor release. These differences may be closely linked to factors such as the degree of nerve damage, inflammatory response characteristics, and nerve regeneration process of each model.

### 4.1 Role of P2X7 receptor in the sciatic chronic constriction injury model

Bennett and Xie (1988) first developed the CCI in rats to mimic human NP. The model was made by separating the biceps femoris from the gluteal muscles, fully exposing and isolating the sciatic nerve, and ligating four loose chromic gut sutures with a 1 mm spacing around them. This model is easy to operate surgically, has mechanical and thermal allodynia, and exhibits the clinical features of NP and an inflammatory component (Zhi et al., 2017). In the CCI model, He et al. (2012) discovered that changes in spinal P2X7R levels were consistent with the development of mechanical hyperalgesia and thermal hypersensitivity and that intrathecal injection of the P2X7R antagonist brilliant blue G (BBG) reversed the CCI-induced mechanical hyperalgesia and thermal hypersensitivity. Their results revealed that P2X7 receptors in the spinal cord play a key role in microglial activation, which may have a significant impact on the formation and development of mechanical hyperalgesia and thermal hypersensitivity in the CCI model. According to Jiang et al. (2016), riluzole inhibits microglial activation by reducing P2X7R expression in the spinal dorsal horn, thereby alleviating NP. Similarly, Lin et al. (2018) used an intrathecal injection of dexmedetomidine to reduce CCI-induced mechanical and thermal hyperalgesia in rats, attributing its analgesic effect to its ability to downregulate spinal P2X7R expression and inhibit ERK phosphorylation. Further studies are needed to elucidate the exact molecular mechanism by which dexmedetomidine regulates the P2X7R and ERK signaling pathways. Furthermore, clinical trials are required to evaluate the efficacy and safety of dexmedetomidine in the treatment of NP in humans. Xu et al. (2016) discovered that the P2X7R agonist Bz-ATP promotes microglial P2X7R expression and the release of inflammatory factors IL-1β and IL-18, ultimately inducing pain. Liu et al. (2023) discovered that Echinacoside inhibits microglial overactivation and inflammation via the spinal P2X7R/FKN/CX3CR1 signaling pathway, thereby alleviating peripheral NP. Additionally, Li et al. (2023) discovered that inhibiting P2X7R reduced glutamate levels in the thalamus and cortical regions, whereas glutamate levels in CCI rats were significantly increased. The findings of this study demonstrate that NP has a significant impact on sleep-related neural activity and may establish new targets for developing sleep disorders under chronic pain conditions. As a consequence, modulation of microglial P2X7R is expected to be an innovative therapeutic strategy for pain relief.

### 4.2 Role of P2X7 receptor in the spinal nerve ligation model

Decosterd and Woolf (2000) established an SNL model of peripheral neuropathic pain with significant research significance. This model involves ligating the spinal nerves L5 and L6 and inducing nerve injury. This model is appropriate for mechanical hypersensitivity because mechanical, heat, and cold allodynia occur rapidly and continue for a long time following surgery (Chen and Lu, 2017) (Moriarty et al., 2016). Peripheral nerve injury can cause the release of local neurotrophic factors, neurotransmitters, chemokines, and cytokines, which induce NP by reducing pain receptor activation thresholds (Liao et al., 2022). The synaptic plasticity of spinal dorsal horn neurons in SNL rats is altered (Bittar et al., 2017). Wu Q. et al. (2021) discovered that electroacupuncture (EA) treatment reduced dendritic spine density, inhibited synaptic remodeling, and reduced inflammatory responses, and was associated with reduced P2X7R expression and improved neurobehavior. The P2X7R agonist Bz-ATP reversed the positive effects of EA, which was consistent with increased P2X7R expression. These findings suggest that EA improves NP by inhibiting P2X7R expression, which reduces abnormal dendritic spine/synaptic remodeling and inflammation. However, they only hypothesized that EA exerts analgesic effects by reducing P2X7R expression, and it would be best to use P2X7R inhibitors or siRNA to confirm this mechanism. Yamashita et al. (2021) discovered that cilnidipine effectively inhibited the Ca^2+^ signaling response and IL-1β release triggered by P2X7 receptors in primary cultures of rat microglia. Furthermore, in a rat experimental model simulating NP, an intrathecal injection of cilnidipine effectively reversed the mechanical hyperalgesia caused by nerve injury, a primary symptom of NP.

### 4.3 Role of P2X7 receptor in the diabetic neuropathic pain model

Diabetic neuropathic pain is a major complication of type I and type II diabetes (Calcutt, 2020). The clinical features of DNP include spontaneous pain, allodynia, and hyperalgesia (Sloan et al., 2021). The main models studied include rats and mice induced by streptozotocin and alloxan, mice fed a high-fat diet, diet and nutritional combination-induced models, transgenic models, and gene knockout diabetes models, including Zucker diabetic obese rats, BBZDP/Wor rats with characteristics of type 1 and type 2 diabetes, type 2 hyperinsulinemia diabetic BBZDR/Wor rats, and leptin-deficient mice (Gao and Zheng, 2014). Transient receptor potential vanilloid receptor 1 is an ion channel activated by heat and inflammatory factors and is involved in developing various pains (Dewaker et al., 2023). Chen et al. (2022) discovered that small interfering RNA (siRNA) of P2X7R and inhibitor of P38 significantly reduced TRPV1 expression by inhibiting the PKC/P38/MAPK/NF-κB signaling pathway and inflammatory factors in the dorsal root ganglia (DRG). Intrathecal injection of P2X7 shRNA can reduce the nociceptive response in rats with diabetic neuropathic pain by activating the PKC/P38 MAPK/NF-κB signaling pathway and TRPV1. Diabetic neuropathic pain and major depressive disorder (MDD) are common complications of diabetes that influence each other (Duan-Porter et al., 2016). Guan et al. (2019) discovered that dihydromyricetin reduced P2X7R expression in rats with DNP and MDD, downregulated ERK1/2 pathway activation, and reduced the release of inflammatory factors TNF-α and IL-1β. These effects ultimately alleviated DNP and depressive behavior. Subsequently, Zhan et al. (Zhan et al., 2024) found that shRNA of long non-coding RNA MSTRG.81401 alleviated pain and depressive-like behavior in diabetic rats with DNP and MDD comorbidity by inhibiting the hippocampal P2X7R-mediated P2X7R/NLRP3/caspase1 cell pyroptosis pathway and pro-inflammatory response. However, these studies share a common limitation: Because of the complexity of DNP and MDD, rat models cannot fully simulate human symptoms, capture the psychological and emotional aspects of pain experienced by human patients, and fully replicate the complex cognitive and social impairments of human patients.

### 4.4 Role of P2X7 receptor in the selective spinal nerve injury model

**FIGURE 3:**
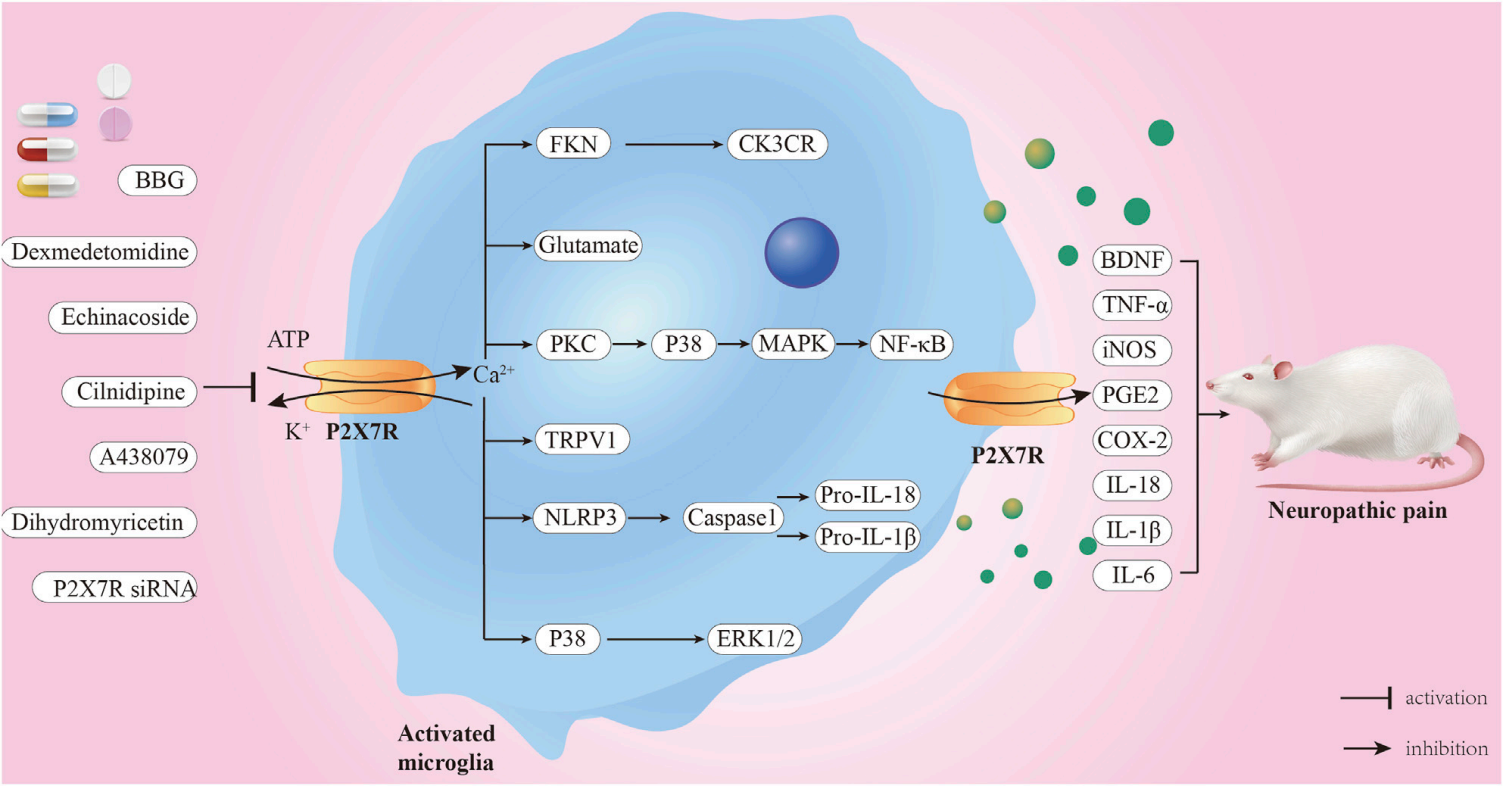
Mechanism of P2X7R-mediated neuropathic pain. When ATP concentration exceeds 100 μM, P2X7R is activated, opening the cell membrane-bound ion channels, leading to Ca^2+^ influx and K^+^ efflux. Consequently, multiple signaling pathways are activated, and inflammatory factors are released, ultimately leading to NP. P2X7R inhibitors and some drugs, including BBG and A438079, can alleviate NP through these pathways.

Decosterd and Woolf (2000) established the SNI model of neuropathic pain in 2000. The SNI model exhibits good mechanical and thermal allodynia, and the duration is longer than other NP models. However, the heat threshold does not change significantly. It is the model closest to clinical neuropathic pain and is suitable for studying the mechanism of clinical treatment strategies for NP (Boccella et al., 2018). Hu et al. (2020) discovered that microinfusion of a P2X7R antagonist (A-438079) into the amygdala of rats significantly reduced the increase in ionized calcium-binding adaptor molecule-1 and glial fibrillary acidic protein (GFAP) immunoreactivity in microglia and astrocytes following SNI. Additionally, A-438079 significantly reduced the decrease in growth-associated protein-43 expression in the spinal cord caused by SNI. However, when A-438079 and a P2X7R agonist (BzATP) were administered simultaneously, all of the effects of A-438079 were reversed. This study suggests that inhibiting P2X7R in the amygdala can protect against NP-related symptoms. This effect may be associated with its inhibition of active microglia and astrocytes in the spinal cord. Zhou et al. (2023) discovered that after SNI, P2X7R expression in the dorsal horn of the rat spinal cord increased over time. Continuous intrathecal injection of the cannabinoid receptor 2 (CB2R) agonist PM226A resulted in a significant decrease in P2X7 protein levels. Furthermore, administering the P2X7R inhibitor A-438079 reduced the symptoms of mechanical hyperalgesia in rats, the number of microglia, and P2X7R expression. These findings suggest that P2X7R is crucial in the neuroprotective process triggered by CB2R activation (Figure 3).

## 5 P2X7 receptor may be a therapeutic target for neuropathic pain

### 5.1 Pharmacological treatments

The intrinsic connection between P2X7R and NP has been well established; consequently, using P2X7R antagonists or inhibitors has the potential to relieve pain and treat NP. As the most extensively studied isoform in drug development, many potent and selective (mainly allosteric) antagonists of P2X7R have been discovered (Illes et al., 2021). However, the development of P2X7R antagonists faces several challenges. These include a lack of selective agonists, such as Bz-ATP, which efficiently activates both P2X1R and P2X3R; differences in sensitivity of mouse, rat, and human homologs to agonists/antagonists; and the fact that various *in vitro* test systems, such as Ca^2+^ influx, Yo-Pro uptake, and membrane current measurements, often yield different results when the same P2X7R ligand is used (Donnelly-Roberts et al., 2009). The P2X7R antagonist A-438079 regulates the TRPV1 receptor by binding to the P2X7R, reducing p38 and ERK1/2 phosphorylation levels, thereby effectively reducing the release of inflammatory factors and thus relieving diabetic NP (Wang et al., 2021). Reports indicate that using P2X7R inhibitors resveratrol (Xie et al., 2017) and riluzole (Jiang et al., 2016) can inhibit the overexpression of P2X7R and GFAP in dorsal root ganglion glial cells, reduce ERK1/2 phosphorylation, and relieve hyperalgesia. The activation of P2X7R opens ion channels on the cell membrane, enhances information transmission between neurons and satellite glial cells, and thus exacerbates pain. Using P2X7R antagonists, such as A740003, can reduce this intercellular communication, thereby alleviating inflammatory and pathological pain (Lemes et al., 2018). P2X7 receptor antagonists, such as A438079, can reduce the release of reactive oxygen species in neurons, reduce DNA damage, and alleviate nociceptive pain (Munoz et al., 2017). In addition, using P2X7R antagonists, such as A839977, JNJ-42253432, and AZ11645373, can inhibit glial cell activation and reduce abnormal discharges of neurons and the central nervous system, thereby effectively relieving pain (Honore et al., 2009) (Lord et al., 2014) (Kobayashi et al., 2023). Several clinical trials of P2X7R antagonists have failed owing to design defects or insufficient validation, resulting in side effects such as headaches, back pain, and fatigue (Timmers et al., 2018) (Platania et al., 2022). Some P2X7R antagonists may be non-specific and interact with other receptors or signaling pathways, thus affecting their efficacy and safety (El Idrissi et al., 2024). Long-term use of P2X7R antagonists may lead to side effects such as immune system dysfunction and abnormal cell metabolism (Zhang et al., 2020). Furthermore, the efficacy of P2X7R antagonists may vary in different types of NP models, which may be related to the specific mechanism of pain and receptor expression levels (Calzaferri et al., 2020). Overall, these findings suggest that P2X7R antagonists have potential for translational applications in pain management; however, further research and development are required.

In addition to specific P2X7R antagonists or inhibitors, some clinically approved specific compounds have been demonstrated to relieve pain by inhibiting P2X7R. Gallic acid, a traditional Chinese medicine ingredient, binds to P2X7R and inhibits the activation of the NF-κB and signal transducer and activator of transcription 3 signaling pathways triggered by P2X7R. This reduces the production of TNF-α and exhibits a significant inhibitory effect on CCI-induced pain (Yang et al., 2021). Trimethoxyflavanone, a naringenin derivative, reduces paclitaxel-enhanced P2X7R expression in the DRG, thereby reducing DRG neuronal sensitization and abnormal spinal glial cell activation, ultimately alleviating pain and neurotoxicity associated with chemotherapy-induced peripheral neuropathy (Mei et al., 2023). Botulinum toxin type A (BoNT/A) also downregulates P2X7R expression and induces microglia to transition from the pro-inflammatory M1 type to the anti-inflammatory M2 type, thereby increasing the pain threshold of rats (Gui et al., 2020). Cilnidipine, a calcium channel blocker, can reduce NP by inhibiting the activation of the P2X7R/IL-1β signaling pathway in spinal microglia [101]. Moreover, dihydromyricetin and palmatine relieve DNP and MDD by reducing the expression of P2X7R in the DRG, spinal cord, and hippocampus, suggesting that they could be effective new drugs for treating patients with DNP and MDD (Guan et al., 2019) (Shen et al., 2018) (Table 1). Palmatine reduced the pain caused by infraorbital nerve injury in the facial trigeminal neuralgia model by inhibiting inflammatory factor release and reducing P2X7R and p38 expression levels (Yin et al., 2021). These findings highlight the possibility of leveraging existing drug resources to accelerate the discovery and development of new and effective analgesics.

**TABLE 1:** Compounds related to P2X7R used in neuropathic pain.

Name	Chemical formula	Species	Models	Observed result	References
A438079	C_13_H_9_Cl_2_N_5_	Rat	DNP	Analgesic effect in NP in rat	Wang et al. (2021)
A740003	C_26_H_30_N_6_O_3_	Rat	Formalin pain	Effective in the formalin pain mode	Lemes et al. (2018)
A839977	C_19_H_14_Cl_2_N_6_O	Mice	CFA	Reduction of thermal hyperalgesia	Honore et al. (2009)
AZ11645373	C_24_H_21_N_3_O_5_S	Rat	CCI	Inhibition of the expression of microglia and inflammatory factors	Kobayashi et al. (2023)
Resveratrol	C_14_H_12_O_3_	Rat	CCI	Inhibition of NP transmission	Xie et al. (2017)
Riluzole	C_8_H_5_F_3_N_2_OS	Rat	CCI	Inhibition of microglial activation and NP	Jiang et al. (2016)
Gallic Acid	C_7_H_6_O_5_	Rat	CCI	Analgesic effect in NP in rat	Yang et al. (2021)
Trimethoxyflavanone	C_18_H_16_O_5_	Mice	PIP	Combatting CIPN-associated pain and neurotoxicity	Mei et al. (2023)
BoNT-A	-	Rat	CCI	Promotion of microglial M2 polarization	Xie et al. (2017)
Cilnidipine	C_27_H_28_N_2_O_7_	Rat	CCI	Inhibition of inflammatory responses and NP	Yamashita et al. (2021)
Dihydromyricetin	C_15_H_12_O_8_	Rat	DNP	Relief of DNP and depression comorbidity	Guan et al. (2019)
Palmatine	C_21_H_22_NO_4_+	Rat	DNP	Relief of DNP and depression comorbidity	Shen et al. (2018)

DNP, diabetic neuropathic pain; NP, neuropathic pain; CFA, complete Freund’s adjuvant; PIP, Paclitaxel-induced pain; CIPN, chemotherapy-induced peripheral neuropathy.

### 5.2 Non-pharmacological and new alternative treatments

Electroacupuncture is an effective treatment for NP. It is widely used internationally owing to its significant therapeutic effect and low risk of side effects. Spinal microglia are important targets of EA in the analgesic effect of tactile allodynia and thermal hyperalgesia (Shan et al., 2007). Electroacupuncture relieves the symptoms of NP by activating the expression of IL-10 in spinal microglia (Ali et al., 2020). Additionally, EA improves synaptic plasticity in rat brain neurons after nerve injury (Xu et al., 2013). Xu et al. (2016) discovered that EA treatment alleviated tactile allodynia and thermal hyperalgesia caused by nerve injury by inhibiting the overexpression of IL-1β mediated by P2X7R-positive microglia. This is consistent with the study of Wu Q. et al. (2021), which revealed that EA reduced dendritic spine density, inhibited synaptic reconstruction, and reduced inflammatory response via P2X7R, thereby reducing NP. Consequently, spinal P2X7R-positive microglia may be a promising target for EA treatment of NP.

Spinal cord epidural stimulation (SCS) is a treatment approved by the US Food and Drug Administration for pain management when pharmacological interventions fail (Attal and Bouhassira, 2019). Compared with conventional SCS, high-frequency SCS exhibits a different analgesic mechanism, as evidenced by a lack of dorsal column activation, weaker inhibition of wide dynamic range neurons in the deep dorsal horn, and a slower onset of pain inhibition (Joosten and Franken, 2020). Kaiso is a transcription factor that regulates immune responses in many tissues. Yu et al. (2024) first proposed the concept of the Kaiso-P2X7R pathological axis and discovered that 10 kHz high-frequency SCS significantly reduced immune responses in the dorsal horn of the spinal cord by inactivating the Kaiso-P2X7R pathological axis in microglia. This resulted in lasting pain relief. Targeting Kaiso-P2X7R in microglia significantly improved the efficacy of conventional SCS treatment, thereby reducing neuroinflammation and providing lasting pain relief. Moreover, DRG and spinal cord dorsal horn cells exhibit high P2X7R expression. Using cell technology to transplant cells such as neural stem cells and olfactory ensheathing cells to the site of sciatic nerve injury can reduce P2X7R expression, thereby relieving pain (Zhang et al., 2019) (Du et al., 2019). Consequently, cell transplantation may become a viable treatment option for nerve injury and pain.

## 6 Conclusion

Approximately 500 million people worldwide suffer from NP, which not only causes severe physical and mental pain but also imposes a significant burden on their socioeconomic status. However, existing NP treatment approaches are ineffective and exhibit unacceptable side effects. Accordingly, developing effective treatment strategies and drugs for NP is an urgent need. With the ongoing in-depth study of the pathological mechanism of NP, our understanding of NP has significantly improved. P2X7 receptor in microglia plays a key role in the occurrence and development of NP. P2X7R activation polarizes microglia M1, causing neuroinflammation and aggravating NP. Inhibiting the function of P2X7R can regulate the transformation of microglia from M1 to M2 and inhibit NP occurrence and development. Consequently, inhibiting P2X7R activity or reducing its expression may be a novel approach to NP treatment, which deserves further and continuous research. Generally, P2X7R in microglia has the potential to become a new pharmacological target for clinical treatment owing to its unique structural characteristics, functional effects, and application prospects in drug development. The research and clinical application of P2X7R antagonists are still in their early stages, and more clinical trials are required to confirm their long-term efficacy and safety. In-depth interdisciplinary research will allow us to gain a better understanding of the role of microglial P2X7R in the pathogenesis of pain, opening up new avenues for pain treatment and giving patient hope. Through continuous scientific exploration and clinical practice, we anticipate that P2X7R antagonists will play an important role in pain treatment and bring substantial improvements and higher quality of life to patients.
